# Case Report: do heart transplant candidates benefit from mechanically supported revascularization?

**DOI:** 10.3389/fcvm.2023.1169165

**Published:** 2023-06-23

**Authors:** Lukasz Pyka, Janusz Szkodzinski, Jacek Piegza, Malgorzata Swietlińska, Mariusz Gąsior

**Affiliations:** ^1^ 3rd Department of Cardiology, Faculty of Medical Sciences in Zabrze, Medical University of Silesia, Katowice, Poland; ^2^ Department of Cardiology, Scanmed Center of Cardiology, Chorzow, Poland

**Keywords:** heart transplant, revascularisation, congestive heart failure, mechanical circulatory support, outcomes, myocardial recovery

## Abstract

**Introduction:**

Recently published studies suggest that percutaneous coronary intervention (PCI) has no significant impact on outcomes in patients with heart failure and stable coronary artery disease. The use of percutaneous mechanical circulatory support is growing, but its value is still uncertain. If large areas of viable myocardium are ischemic, the benefit from revascularization should be evident. In such instances, we should strive for complete revascularization. The use of mechanical circulatory support in such cases is vital because it provides hemodynamic stability throughout the complex procedure.

**Case report:**

We present a case of a 53-year-old male heart transplant candidate with type 1 diabetes mellitus, initially considered unsuitable for revascularization and qualified for heart transplantation, transferred to our center due to acute decompensated heart failure. At this time, the patient had temporary contraindications for heart transplantation. As the patient was considered no-option, we have decided to reassess the possibility of revascularization. The heart team opted for a high-risk mechanically supported PCI with the aim of complete revascularization. A complex multivessel PCI was performed with optimal effect. The patient was weaned off dobutamine on the second day post-PCI. Four months post-discharge, he remains stable, is in NYHA II class, and has no chest pain. Control echocardiography showed improved ejection fraction. The patient is not a heart transplant candidate anymore.

**Conclusions:**

This case report shows that we must strive for revascularization in select heart failure cases. The outcome of this patient suggests that heart transplant candidates with potentially viable myocardium should be considered for revascularization, especially as the shortage of donors persists. In the most complex coronary anatomy and severe heart failure, mechanical support in the procedure might be essential.

## Introduction

Recently published studies suggest that percutaneous coronary intervention (PCI) has no significant impact on outcomes in patients with heart failure (HF) and stable coronary artery disease (CAD) (1, 2). In patients with severe CAD and HF, the use of percutaneous mechanical circulatory support (MCS) is growing. However, its impact on outcomes is still uncertain, and, in many instances, the potential benefits are diminished by complications (3–5).

Nonetheless, we believe that a significant population of ischemic HF patients benefits from these interventions, especially when revascularization is achieved in large areas of viable myocardium. In such instances, we should strive for complete revascularization, including interventions on chronically occluded arteries (6, 7). MCS is vital because it provides hemodynamic stability throughout the complex procedure.

## Case report

We present a case of a 53-year-old male heart transplant candidate with type 1 diabetes mellitus who was initially hospitalized at the intensive care unit in our center in March 2022 due to acute decompensated heart failure (ADHF). He was considered unsuitable for revascularization by the heart team, as his severe left ventricular impairment with a left ventricular ejection fraction (LVEF) of 15% and advanced multivessel coronary artery disease were considered extremely high risk and futile both for PCI and coronary artery bypass grafting. The patient was qualified for heart transplantation (OHT) by the heart transplant team. He was treated medically and discharged in stable condition.

In May 2022, the patient was transferred to our cardiology department due to a second ADHF episode, with severe congestion, hypotension, pleural effusion, severe asymmetric lower limb edema, and signs of infection. On admission, his echo showed a LVEF of 18% and an end-diastolic left ventricle dimension of 64 mm, with moderate mitral and tricuspid insufficiencies and good right ventricular function. The patient was stabilized with inotropes, intravenous diuretics, and pleurocentesis. As Doppler ultrasound and CT-angio confirmed lower limb deep vein thrombosis (DVT) with an infected ulceration, the patient was temporarily taken off the OHT list. Subsequently, after initial stabilization on oral medical treatment, he was transferred back to the referring cardiology department for further observation and rehabilitation. He was readmitted after 25 days in deteriorated clinical condition, on dobutamine support, hypotensive, in NYHA III/IV class. At this time, an urgent heart transplant was considered the only option. Therefore, he underwent right heart catheterization (cardiac output 3.55 L/min; cardiac index 1.92 L/min/m^2^; pulmonary vascular resistance 2.25 Wood units). Nonetheless, after the heart transplant team reassessment, he was still deemed unsuitable for OHT (due to persistent significant DVT and unclear infectious status with significantly elevated inflammation markers despite treatment).

At this time, we have decided to reassess the possibility of revascularization, as the patient was considered no-option, dobutamine-dependent, unsuitable for urgent OHT. In our healthcare system, only OHT-qualified patients are potential candidates for long-term mechanical circulatory support; therefore, such treatment was not available. The patient's coronary angiography before initial OHT qualification revealed a critical, calcified proximal left anterior descending artery (LAD) lesion, a chronic total occlusion (CTO) of the circumflex artery (LCx), a significant left main stenosis, and diffuse disease of the right coronary artery (RCA) with a critical lesion at the crux cordis. The heart team opted for a high-risk MCS PCI with the aim of complete revascularization. As revascularization was considered the last viable treatment option, myocardial viability or ischemia testing was not performed.

At the beginning of the procedure, two Abbott Perclose ProGlide devices were inserted after an ultrasound-guided femoral puncture. The Abiomed Impella CP catheter was inserted into the left ventricle. With the single access technique (puncture of the Impella hemostatic sheath), we were able to engage the left coronary artery with an EBU 3.5 catheter and perform a control angiography, which revealed similar coronary artery status to that before OHT qualification. A Pilot 50 guidewire easily crossed the LAD lesion; however, we were unable to introduce the HD IVUS Acist Kodama catheter. After meticulous 2.5-mm × 20-mm NC balloon predilatation, two sirolimus-eluting stents were implanted up to the ostium of the LAD (Ultimaster Tansei 2.5 mm × 28 mm and 3.0 mm × 38 mm). As IVUS at this point revealed stent underexpansion and further postdilatation provided insufficient results, 70 impulses of intravascular lithotripsy (Shockwave 3.5 mm × 15 mm) were performed to very good effect.

Subsequently, the circumflex artery was recanalized using a microcatheter-supported Pilot 150 guidewire and followed by the implantation of three everolimus-eluting stents (Xience Pro 2.25 mm × 23 mm, 2.5 mm × 23 mm, 2.5 mm × 18 mm). TIMI 3 flow was restored with optimal angiographic and IVUS results. Finally, the left main PCI was performed using a provisional technique with implantation of a 4.0-mm × 12-mm sirolimus-eluting Ultimaster Tansei stent, postdilated with a 4.5-mm × 6-mm NC balloon. Optimal angiographic and IVUS results were achieved. The patient remained hemodynamically stable through the procedure. The Impella CP was removed in the cathlab, and the Proglide presuture device provided hemostasis.

After 6 days, the patient was taken back to the cathlab. Coronary angiography showed optimal results of the previous procedure. After predilatation, a PCI of the right coronary artery with the use of three sirolimus-eluting stents was performed (Ultimaster Tansei 3.5 × 38, 2.75 × 24, 2.5 × 33).

**Figure 1:**
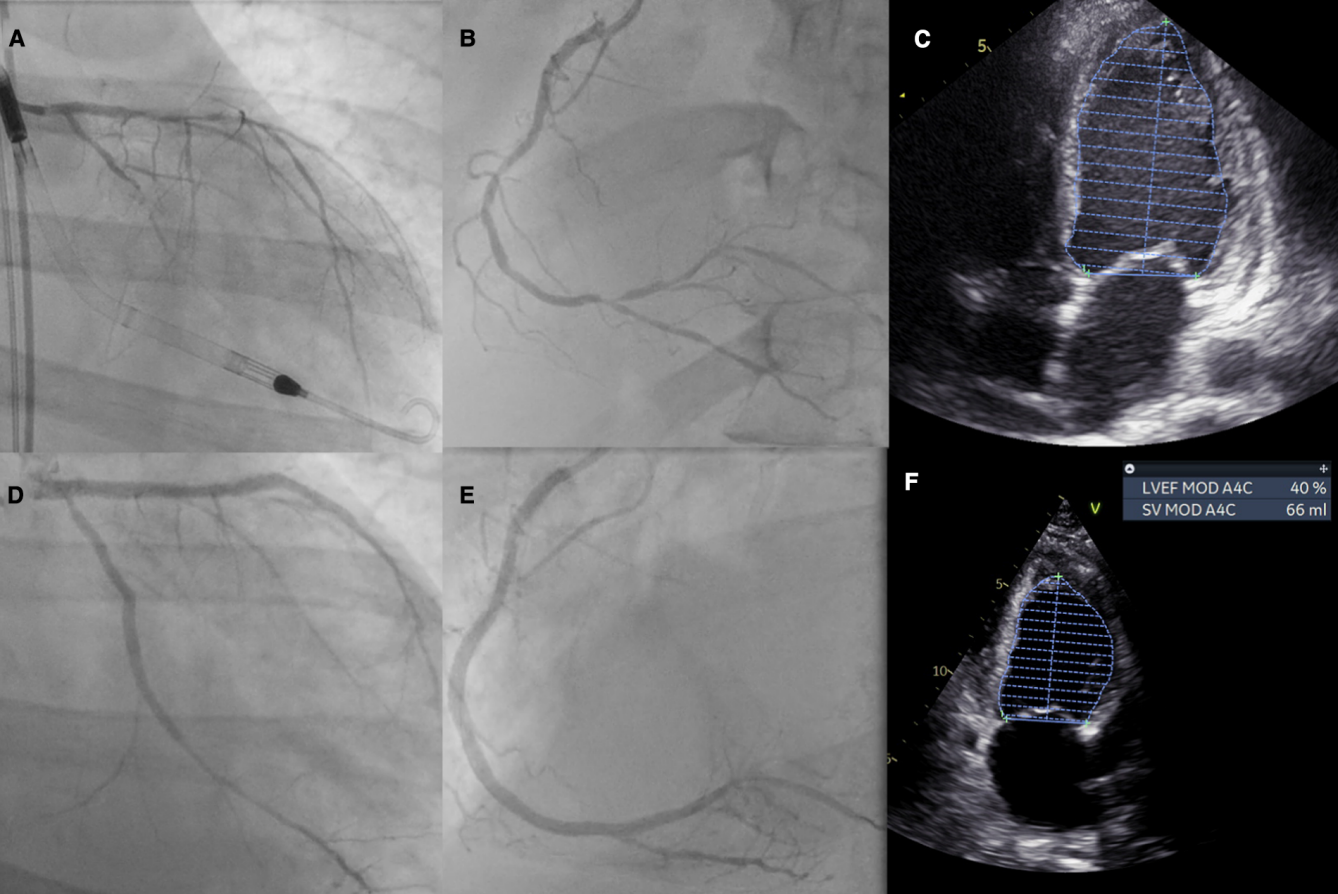
Patient summary: (**A**) initial left coronary artery anatomy with visible Impella support, (**B**) initial right coronary artery anatomy, (**C**) initial echocardiography with an LVEF of 18%, (**D**) final PCI effect of the left coronary artery, (**E**) final PCI effect of the right coronary artery, and (**F**) echocardiography on follow-up with an LVEF of 37%–38%. LVEF, left ventricular ejection fraction.

Initial and follow-up echocardiography is presented in Supplementary Video 1. The angiographic images of the procedures are presented in Supplementary Video 2. The IVUS images are presented in Supplementary Video 3. The summary of initial and final angiography and echocardiography is presented in Figure 1.

The patient was weaned off dobutamine on the second day post-PCI. The in-hospital stay was complicated by pneumonia, which was subsequently treated in the referring cardiology department. Four months post-discharge, he was controlled at the outpatient clinic. The patient remains stable, is in NYHA II class, and has no chest pain. Control echocardiography showed an LVEF of 37% and a mild mitral insufficiency. The patient is currently not an OHT candidate. Persistent DVT, despite antithrombotic treatment, remains the most important clinical problem at the time of follow-up.

## Discussion

In general, it is the policy at our center to utilize all of the available treatment methods for severe HF patient before OHT qualification. This includes almost routine revascularization. In these patients, long-term results are generally satisfactory, especially when complete revascularization is possible (6). There are, however, select cases when the patient is not revascularized. This concerns mostly patients with extremely complicated coronary anatomy and the most severe left ventricular impairment, especially when the benefit of revascularization is doubtful. In such cases, myocardial viability testing may be useful in the decision-making process. However, severe dilatation of the left ventricle and thinning of the myocardium may be considered surrogates for lack of viability. Moreover, in hemodynamically unstable patients, inotrope-dependent, when OHT is considered urgent (in-hospital), revascularization is rarely performed to avoid potential complications or the need for a dual (or triple) antithrombotic regimen. If the heart team decides to perform PCI in OHT candidates, especially in complex coronary anatomy (unprotected left main lesion, multivessel disease, last patent vessel), a periprocedural hemodynamic compromise might be expected. In such cases, MCS should be considered to provide patient stability throughout the procedure.

This case report presents the treatment and outcomes of a patient in whom, at an early stage of treatment, there was a decision to treat CAD conservatively. Such a decision, a premature one in our opinion, complicated the patient's further clinical course. Performing complete revascularization produced left ventricular improvement exceeding expectations, even with no initial proof of myocardial viability. MCS support enabled the operators to perform the procedure safely and optimally. In such complex HF cases, the ever-changing clinical scenario might compel the physicians to challenge initial treatment decisions in the best interest of the patient.

## Conclusions

We have presented a case of a dramatic clinical and LVEF improvement in an OHT candidate with temporary contraindications after complete revascularization. High-risk PCI was the only viable option and provided results exceeding our expectations. This case report shows that in select HF cases, we must strive for revascularization, despite recent trial results. The question remains whether revascularization during initial OHT qualification could have prevented the ADHF episodes. The patient should have never entered the OHT waiting list, especially with the significant comorbidities. The outcome of this patient suggests that OHT candidates with potentially viable myocardium should be considered for revascularization, especially as shortage of donors persists. In the most complex coronary anatomy and severe LVEF impairment, MCS might be essential.

## Data Availability

The datasets presented in this article are not readily available because this is a case report. Requests to access the datasets should be directed to the corresponding author.
